# Annotations

**Published:** 1898-12-10

**Authors:** 


### ANNOTATIONS.

### Partners in Crime.

COMMENTING upon the recent charges of blackmailing by a group of men who have just been committed for trial at the Central Criminal Court, the *Medical News* of New York makes the very pertinent inquiry, What will be done in regard to the dupes? It is understood that a large number of letters containing enclosures of money are in the hands of the police —letters which had been sent in answer to circulars, offering on receipt of two guineas to stop any proceedings which might be taken on account of the writers having purchased certain medicines. The *Medical News* says: "Everyone who sent the two guineas evidently confesses that her idea in taking the miraculous tabules was to commit what . . . . is a felony before the English law. Will the letters be used to prove this, and the confessed criminals be brought to justice; or will the police compound a felony and permit the affair to drop out of sight? In other words, does the statute on the English statute book mean anything or nothing?" We do not think that the matter is quite so simple as our American contemporary imagines. Still, what is quite clear is that some attempt should be made to punish both parties to an attempted crime; and if it is true that the prisoners in this case are guilty of the crime imputed to them, a matter as to which we express no opinion, it seems wrong that all these women, who have shown themselves so ready to commit an act which is a felony, should receive no warning of the proper consequences of their recklessness. Everyone will agree with the very severe remarks made by Mr. Alderman Newton upon the conduct of the "newspapers in which these rank advertisements luxuriate only to produce any appalling crop of pruriency and crime." But the suppression of advertisements is only the first step towards the diminution of criminal abortion. Not only should such advertisements be suppressed, and the dealers in abortifacients be punished, but women should be taught that in accepting the medicines so offered they are making themselves partners in crime, and liable to become sharers in punishment.

### The School Board and the Truants.

WITH an educational code which gives every child the opportunity of getting all the necessary elements of education, we still find a surprising proportion of the boys and girls of the poorer parts of our towns growing up in almost complete illiteracy. In London, out of a total of 529,000 children of school age, there are 98,000 perpetually absent, the average attendance being only 431,000. Many of these children play truant, and their parents connive at their truancy. As soon as the children are able to earn a little, no matter how trifling the sum may be, there are parents who will keep them from school, and set them to such jobs as they can get for the sake of the few shillings that they earn. One cannot but pity the poverty of the parents, above all when it is the case of a widowed mother, and not, as too often happens, a hard-working woman supporting, with the help of her children, a lazy husband. But it is not fair to let their needs interfere with the children's rights. Children growing up as illiterate as their parents have no prospect in life but the hopeless, ill-paid monotony of unskilled labour; they should, therefore, for their own sake, be kept at school till they have mastered the essentials of knowledge. The law punishes parents who fail to send their children to school, but till lately the punishment inflicted in London was not sufficiently heavy to prevent the commission of the offence. The neglectful parents were fined five shillings, and in the event of their still failing to send their children to school they might, at the expiration of two months, be summoned and fined again. Now, the children do not earn a great sum, and doubtless the mother feels sorely aggrieved that this fine should deprive her of five shillings of it every two months; but, after all, it pays her to keep the child from school and send him or her to work, even with so heavy a tax to pay for the privilege; if there remained but another five shillings after it was paid she would think it worth while to defy the law for that. That is the parent's point of view, who cannot see any good to be gained from education; but it is not desirable that it should be the public's. Nevertheless, we fear that many people, looking only to the parents' immediate comfort, will sympathise with their attempts to evade the requirements of the law.

### The Metropolitan Provident Medical Association.

A LETTER appears in the *Times* advocating the claims of the Metropolitan Provident Medical Association. The object of this association is "to establish provident dispensaries in London and the suburbs, and thus to enable working people to obtain efficient treatment for themselves and their families, through their own thrift and self-reliance, on the method of insurance, by means of small regular payments." We need hardly say that with the principle here laid down we have every sympathy, looking as we do upon the provident or insurance principle as the only one on which the poorer of the wage-earning population can pay as they should pay for medical attendance. The letter is signed by names which should inspire confidence, and the movement is and has been supported by many who have shown much interest in the problem it is meant to solve. Yet notwithstanding the support of philanthropists and the general sympathy with which the scheme has for so many years received, it makes but little headway, for at the end of 20 years' work there is only a total members' contribution of about £5,000 a year, which does not seem much in a great place like London. The establishment in every part of London of provident dispensaries by which the gap between private practice and the hospitals may be properly filled up is a matter of great importance. But if the fees are to be calculated on what is really a charity scale (which a penny a week is, seeing that the doctor cannot live by it), the greatest pains must be taken to keep out all who are able to pay those ordinary fees on which experience teaches that a medical man can make a living. We fear that the provident principle will not develop its full utility until it is so organised that medical men shall feel that it is an honour to be on the staff of a provident dispensary. That day has not come yet, but a little organisation and honest co-operation among medical men might do much to establish such institutions on a proper basis.

